# Editorial: Implementing mental health prevention and promotion programs: a sustainable approach, volume II

**DOI:** 10.3389/fpsyg.2026.1727833

**Published:** 2026-03-27

**Authors:** Carla M. Santos de Carvalho, Santiago Gascón-Santos, Adrian Alacreu-Crespo, Jorge L. Ordoñez-Carrasco

**Affiliations:** 1 University of Coimbra, CINEICC, Faculty of Psychology and Educational Sciences, Coimbra, Portugal; 2 Universidad de Zaragoza, Zaragoza, Spain

**Keywords:** education, health system, mental health promotion, prevention, suicide

Promoting mental health is a concept that goes beyond prevention; it requires individuals and their communities to take an interest in creating the means and conditions for maintaining healthy lifestyles (Gascón-Santos et al., 2025). Alternatives to various mental health issues have been proposed in different areas with positive results, although the challenge is to ensure that these actions are sustained over time so that their effectiveness can be evaluated (Kalra et al., 2012). This second volume on “Advancing Sustainable Mental Health Prevention and Promotion Programmes” seeks to compile relevant experiences in health promotion in the health sector, at different educational levels and in organizations.

The integration of promotion into public health systems is considered the cornerstone of sustainability. Sangwan et al. present the results of a study to increase the capacity of primary care health professionals in Rajasthan (India), showing the benefits of having standardized tools for the detection of mental disorders. Along these lines, Sánchez-Recio et al., analyse the moderating effects of one of the variables that has shown unequivocal relationships with health—social support—concluding that primary care should promote intervention strategies that foster social networks as a key element in maintaining and improving the health of the population. There are numerous sources of information used in primary care to detect possible mental health disorders at an early stage. As an example, we describe the findings of Yang et al., who link body mass index to the risk of depression, and show that this relationship is mediated by blood glucose levels. The study of Chen et al. analyzed data from 8,748 participants in the NHANES study (2017–2023) to examine the association between the A Body Shape Index (ABSI) and depression, while also exploring fasting blood glucose (FBG) as a potential mediating factor. Fully adjusted models revealed a significant positive relationship between ABSI and depression, an effect that was particularly evident among individuals with less than some college education. Mediation analyses further showed that FBG partially explained this association, accounting for 15.8% of the total effect. These findings suggest that higher ABSI values may be linked to an increased risk of depression, partly through metabolic pathways reflected in fasting glucose levels.

Beyond the realm of healthcare professionals, this volume addresses the ability to train lay people to contribute to mental health promotion. Scheele et al., discuss the benefits of first aid programmes for detecting signs of emotional problems. At the same time, Shekhar et al. present the results of HR-led training to improve wellbeing in the workplace, which produced significant improvements in employees, demonstrating that professionals without clinical experience can generate lasting positive interventions.

However, it is perhaps in the field of education that the greatest scientific output can be observed in terms of mental health assessment and intervention, both in primary and secondary education and at university level, given the worrying figures for bullying and suicide attempts, among other issues, recorded in this sector. Of particular note is the evaluation of an emotional learning programme carried out with primary school children by Antunes et al., whose results support the importance of incorporating knowledge of emotions from an early age. Indeed, there are many potential risks present in the school population and countless variables involved, making it essential to maintain an overview of the studies carried out in this field. One of the most pressing problems is that of bullying and its harmful consequences. Jiang and Chai, focusing on the last years of primary education and the first years of secondary education, explore the relationship between bullying and non-suicidal self-harm and the role of depression and social support, concluding that there is a moderate mediating effect between bullying and non-suicidal self-harm in students, that depression is a mediating variable in this relationship, and that social support mitigates the effect of self-harm. Complementing these data, Anan et al., report positive correlations between bullying, negative affect, sleep quality, and non-suicidal self-harm. On the other hand, focusing on secondary education, Zhang and Chen, studied the mediating role of self-efficacy in learning in the relationship between wellbeing and academic performance. Given that academic pressure can significantly affect mental health and overall wellbeing, the results highlight the importance of including and maintaining specific interventions to improve mental health and self-efficacy in learning among young people.

Remaining within the educational sphere, but focusing on the university level, Wang et al., analyse the relationship between exposure to online risks and depression, concluding that greater exposure to risk can directly and indirectly lead to depression by increasing emotional insecurity and suppressing happiness, along with other knock-on effects. This type of risk, which affects all age groups, is most evident in young people, which is why Yuan et al., examine the background and underlying mechanisms of cyberbullying among university students in light of social comparison theory (SCT). They found that upward social comparison was positively associated with cyberbullying, mediated by relative cognitive and emotional deprivation, while belief in a just world (BJW) negatively moderated the relationship between relative deprivation and cyberbullying.

Valuable examples of other research aimed at exploring the contributions of new technologies to the mental health of university students include studies such as that by Zhan (2025), who, based on cognitive-behavioral therapy and self-management theories, investigated the effectiveness of an intelligent “chatbot” in providing support to students in self-managing anxiety, achieving a significant reduction in anxiety symptoms. The effectiveness of this “chatbot” was mediated by cognitive restructuring, behavioral activation, and emotional regulation pathways. The study is interesting in that it highlights the potential of AI as a tool for anxiety management, while identifying areas for future research, including long-term effectiveness and cross-cultural applicability. Along the same lines, Hao et al., explored the mechanisms of AI-assisted psychological intervention in university students and observed a significant correlation between the frequency of technology use and negative psychological indicators, providing a theoretical and practical basis for creating mental health support systems in higher education.

Seeking to contribute to knowledge about student wellbeing, An et al., confirmed that cognitive avoidance, perfectionism, stress, and rumination were positively correlated and identified three indirect mediating pathways: the effect of perfectionism, the effect of stress, and a chain effect through both. On the other hand, two studies analyzed the effects of literacy on young people's mental health. Shanshan et al., found that physical literacy was positively correlated with mental health and resilience, while, on the other hand, Shi and Tian, concluded that mental health literacy positively predicted attitudes toward seeking psychological help, which would provide a theoretical basis and specific strategies for optimizing psychological services in university students.

With regard to prevention and mental health promotion, the main themes of this volume, other sectors such as the elderly and people with disabilities have been receiving special attention in recent years. The role of unwanted loneliness is being widely studied, not only in the elderly population, but in all age groups. Zeas-Sigüenza et al. propose a model of loneliness spectrum and a systemic intervention framework that focuses on structural determinants and the prevention of isolation as a fundamental public health strategy. On the same topic, the findings of Hernández-Díaz et al., underscore the importance of addressing loneliness as a public health priority, paying special attention to vulnerable groups and developing prevention, detection and intervention strategies tailored to different age groups, which could be implemented through primary care and educational institutions.

Regarding the promotion of mental health in people with disabilities, Rong et al., verified the mediating effect of self-perceived burden between meaning of life and dignity in disabled patients. On the other hand, Usman et al., highlight the existence of barriers to accessing mental, sexual, and reproductive health services for people with psychosocial disabilities in Nigeria, emphasizing some false cultural beliefs.

Finally, considering that one of the most dramatic consequences of mental health loss is suicidal ideation, various studies on this issue are addressed. Sanz-Gómez et al., analyzed the predictive validity of the SAD PERSON and NO HOPE scales. While the former showed high specificity and low sensitivity in predicting suicide risk, the latter had low sensitivity but high specificity. An improved predictive model incorporating key variables from both scales demonstrated greater sensitivity and specificity. For their part, Yang et al., conducted a comprehensive review of factors related to suicide among nursing staff, concluding that the suicide rate in this sector exceeds that of the general population due to a higher incidence of emotional problems and workplace bullying. They conclude that the suicide rate can be reduced through primary and tertiary prevention. A third study, original in terms of its subject matter, is that of Chen, Li et al., which assesses the risk and protective factors associated with suicidal ideation in infertile couples. Fertility-related pressure, anxiety, depression, and marital quality were associated with suicidal ideation in both men and women, while greater resilience mitigated this risk in women.

Conclusión: estas y muchas otras experiencias en la promoción de la salud mental en diversos medios aún se encuentran en una fase incipiente, pero todas ellas comparten la característica común de atribuir una parte de la responsabilidad al usuario y a la comunidad, más aún en países en los que se observa falta de profesionales, o colapso de los servicios de salud mental. La diversidad de poblaciones y metodologías expuestas en este volumen, lejos de presentar un panorama fragmentado de las intervenciones, sirve para poner de relieve el enorme número de variables que intervienen en el mantenimiento de la salud, así como la necesidad de diseñar programas holísticos que tengan en cuenta aquellos componentes que hayan mostrado indicios significativos de mejora y de sostenibilidad económica y temporal.
